# Qualitative Study of General Public Views towards Adverse Drug Reactions in Lithuania

**DOI:** 10.3390/healthcare9030303

**Published:** 2021-03-09

**Authors:** Agne Valinciute-Jankauskiene, Kubiliene Loreta

**Affiliations:** Department of Drug Technology and Social Pharmacy, Lithuanian University of Health Sciences, Sukileliu Ave. 13, LT-50166 Kaunas, Lithuania; loreta.kubiliene@lsmu.lt

**Keywords:** adverse drug reaction, consumers, reporting, pharmacovigilance

## Abstract

Direct patient reporting of adverse drug reactions (ADRs) is available in many countries, as patients are often knowledgeable about their health conditions and medicines. This study aimed to assess whether patients can recognize ADRs and whether they know how to proceed with ADR reporting. The study also assesses ADR information sources and the main barriers to reporting. Through the purposive and snowball sampling techniques, 42 consumers participated in focus group discussions. All discussions were audio recorded, transcribed verbatim, and analyzed for thematic content analysis. The thematic content analysis yielded four major themes: knowledge about medication safety, ADRs, and pharmacovigilance; information sources about medicines and ADRs; knowledge about ADR reporting; attitudes towards ADR reporting; benefits of ADR reporting; barriers to ADR reporting. Participants were able to identify ADRs and used different information sources about medicines and ADRs to confirm their beliefs. However, the poor communication between consumers, pharmacists, and physicians is the main barrier to ADR reporting. This study identified the challenges in relation to pharmacovigilance in Lithuania from patients’ perspectives. Our study indicated a lack of clearly set standards and communication guidelines between patients, physicians, and pharmacists. Active pharmacovigilance might help develop consumer habits regarding the reporting of ADRs in the presence of spontaneous pharmacovigilance.

## 1. Introduction

Spontaneous reporting of adverse drug reactions (ADRs) is the most extensively used method for the post-marketing safety surveillance of medicines. It involves various stakeholders, including competent authorities, marketing authorization holders, and healthcare professionals. Before 2012, some European Union (EU) countries included patient participation in pharmacovigilance by allowing direct reporting by patients (e.g., Denmark in 2003, the Netherlands in 2003) [1]. Since 2012, it has been mandatory (by law) for countries in the EU to provide an opportunity for patients to report suspected ADRs directly to the national competent authority [2]. The benefits of direct patient reporting of ADRs have been revealed by a number of studies [3,4,5]. Most found that direct reporting makes positive contributions in ways that differ from the perspectives of healthcare professionals and leads to the detection of new drug safety signals [6,7,8].

In Lithuania, the State Medicines Control Agency under the Ministry of Health of the Republic of Lithuania (SMCA) is responsible for the management of the spontaneous ADR reporting system. Healthcare professionals, pharmacists, and marketing authorization holders are not the only groups that can report ADRs directly to the SMCA. Since 2013, patients have been able to report ADRs directly using an online form on the SMCA webpage, which can be downloaded and sent via email, post, or fax after completion. It can also be completed during a call to a dedicated phone number [9,10]. No personalized feedback is sent to the reporter unless additional information is required.

Twenty patients took advantage of the opportunity to report ADRs to the SMCA in 2013. The number of reports received from patients fluctuated from 2013 to 2020, but did not change significantly and remained generally low. One study found that the number of ADR reports received does not reflect the number of drug reactions, especially considering that the number of medications used in Lithuania increased during the same period [11].

Patients are often knowledgeable about their health conditions and medicines. However, poor awareness, confusion, and difficulties related to ADR reporting procedures and forms result in the under-reporting of ADRs [12]. To improve voluntary ADR reporting by patients, it is vital to identify factors that affect the numbers of received reports. We aimed to assess whether patients recognized ADRs and knew how to proceed if an ADR occurred. We also assessed the ADR information sources and main barriers to reporting. The gaps identified should help us to understand the full potential and problems associated with patient reporting.

## 2. Materials and Methods

### 2.1. Ethical Approval

This study was approved by the Kaunas Regional Biomedical Research Ethics Committee, Lithuania (reference number BE-2-59). All participants provided signed informed consent.

### 2.2. Study Design

A focus group (FG) approach was used for this study. The method aimed to obtain data from a purposely selected group of individuals rather than from a statistically representative sample of the broader population. During group discussions, group members were encouraged to communicate with one another and to exchange ideas and comments about members’ experiences and viewpoints. We aimed to gain an in-depth understanding of social issues and explanations for behaviors in a way that would be less easily accessible from responses obtained by direct questioning of individuals [13].

### 2.3. Study Sampling and Recruitment

Targeted participants were recruited using a combination of purposive and snowball sampling through contacts made by the researchers. Participants were collected through contact with various associations for older people, from whom recommendations were also received for further group discussions. The number of required FG sessions was not predetermined. However, the data collection continued until it seemed to reach a saturation point, at which there was a repetition of themes and no new information was shared [14]. The recruited participants were selected using predetermined inclusion and exclusion criteria. The inclusion criteria to participate in the discussions were that a participant must be >18 years of age and sign an informed consent form. Participants who did not understand or speak Lithuanian and who declined to participate were excluded from the study.

### 2.4. Data Collection

**Figure 1:**
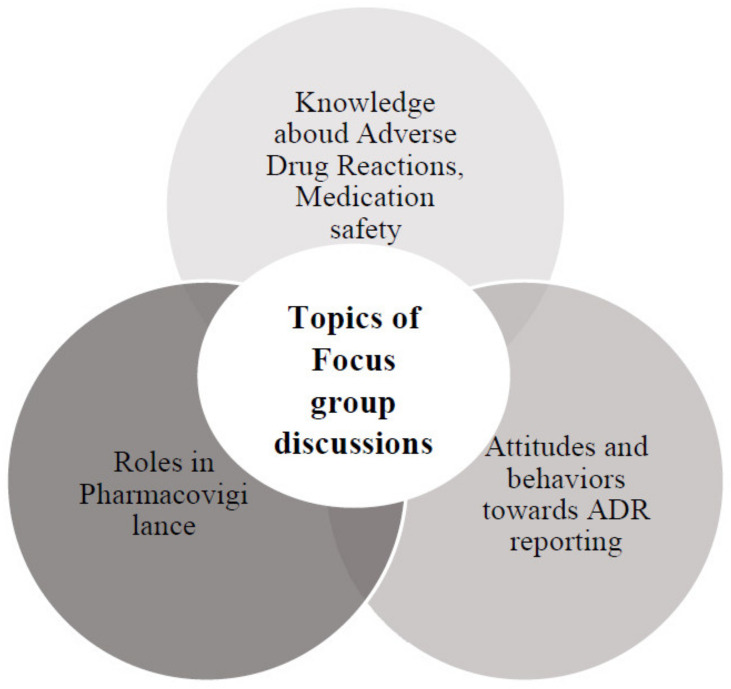
Summary of topic guides for focus group discussions.

FG discussions were performed from December 2019–October 2020. Before starting a discussion, the moderator used an explanatory statement to describe the study objectives. Written informed consent was then obtained from each participant. Every participant was informed that the interview would be recorded. The moderator assured that confidentiality and anonymity would be strictly maintained. A question guide was developed after reviewing previous studies on ADR reporting [15,16]. A summary of the topic guides used for the FG discussions is presented in Figure 1.

All discussions were conducted by the researcher (first author) and were audio-recorded. The group discussions were 45 min to 1 h. Discussions were carried out until saturation was reached. No new themes emerged after three discussions, but one further FG discussion was conducted to confirm that saturation was achieved and assess whether any new themes had emerged [14].

### 2.5. Data Analysis

All audio recordings were transcribed verbatim and their content thematically analyzed using a previously described method [17]. The first step included a review of the data collected. Initial codes from the transcripts were then generated. These identified codes were categorized thematically and by color to bring clarity to the coding process. The themes were reviewed and confirmed to ensure internal homogeneity and external heterogeneity. They were then defined and named, and a report was prepared.

## 3. Results

### 3.1. Demographic Characteristics of Participants

**Figure 2:**
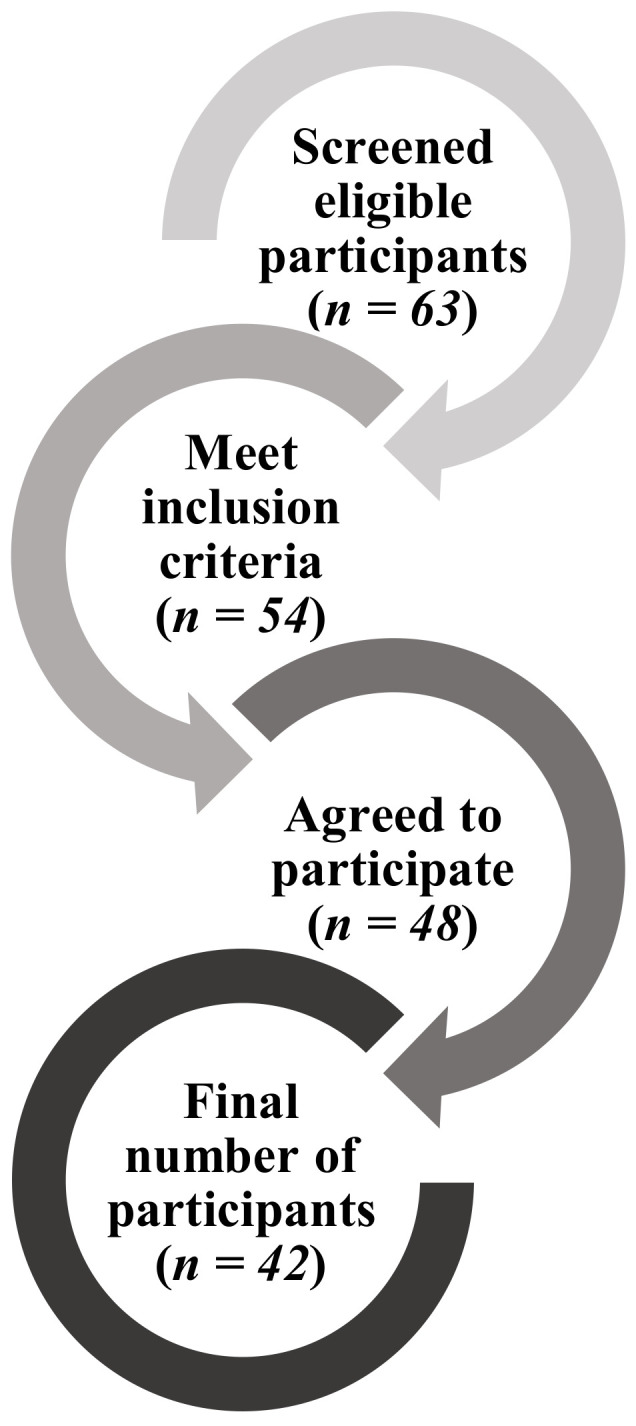
Flow diagram of participant recruitment for focus group discussions.

Forty-two participants (29–90 years of age) were included in the study. A flow diagram of participant recruitment is presented in Figure 2. 

About 31% (*n* = 13) of the participants were male and 69% (*n* = 29) were female. The results for the demographic distribution of the participants among the FGs are presented in Table 1.

### 3.2. Thematic Analysis of Content

The thematic analysis of the FG discussions revealed six major themes: (1) Knowledge (1) knowledge about medication safety, ADRs, and pharmacovigilance; (2) information sources about medicines and ADRs; (3) knowledge about ADR reporting; (4) attitudes towards ADR reporting; (5) benefits of ADR reporting; (6) barriers to ADR reporting.

#### 3.2.1. Theme 1: Knowledge about Medication Safety, ADRs, and Pharmacovigilance

##### Subtheme 1: Knowledge about Definitions

Most participants knew and could generally explain the safe use of medicines, ADRs, and self-medication. They could give comprehensive definitions of these terms in their own words. ADRs were described using terms that included types of allergies, dizziness, and gastrointestinal disorders. However, some participants indicated safe use of medicines as *“Everyone’s personal understanding of how to use medicines”* (FG2). 

None of the participants were familiar with the term “pharmacovigilance”.

##### Subtheme 2: Memories about ADRs

Participants in all discussion groups had vivid memories about ADRs they or family members experienced, even if the ADRs occurred some years ago. All were able to describe at least one ADR episode. During each group discussion, a large part of the time was spent sharing memories and experiences of their own or family members’ ADRs: “*And I had such a reaction, I almost died*” (FG1); “*I can tell not my case, but my husband’s*” (FG3).

##### Subtheme 3: Self-Medication Practices

During discussions, participants were asked to describe the safe use of medicines and how personal self-medication practice issues have evolved. The participants understood and highlighted the importance of compliance with instructions provided by doctors and pharmacists. However, during discussions, participants confessed that they did not always, or sometimes did not even often, comply with usage instructions: “*You just go and buy, you know what you need*” (FG1); “*I use* (medicines) *sometimes responsibly, sometimes irresponsibly*” (FG1).

##### Subtheme 4: Causes of ADRs

When participants were asked about causes of ADRs, the responses included food, lifestyle, stress, and self-medication as factors. Some participants responded that the specialists prescribing the medicines were responsible for ADR incidents. During discussions, other participants mentioned psychological factors related to ADR occurrence. The participants highlighted that beliefs about treatment are important for efficacy: “*You know, this is fate and destiny. If you believe, the medicine will suit you*” (FG1); “*Yes, you must have the mind-set that you will be okay with that pill today*” (FG1).

#### 3.2.2. Theme 2: Information Sources about Medicines and ADRs

Participants indicated that the most important information to know about medicines was compatibility with other medicines, usage instructions, and composition, especially if one medicine was replaced by another. Respondents gave a few primary information sources about medicines and ADRs (i.e., physicians, pharmacists, consumer leaflets and the Internet). 

##### Subtheme 1: Pharmacists and Physicians as Information Sources

Greater trust was expressed in pharmacists than in physicians as an information source about medicines. Participants had favorite pharmacists and asked for advice when purchasing medicines from those individuals: “*Pharmacists advise better than physicians*” (FG1); “*Maybe from a physician about the disease, and about medicines, of course, if you are not sure and something is happening, we may get more* (information) *from pharmacists*” (FG3); “*I try to go to the same pharmacist. I have such beloved ones. When I need something, I can ask, and they explain more. I trust my beloved pharmacist more. I am never turned down*” (FG4).

However, one participant described an experience during which neither the physician nor pharmacist provided information about the prescribed medicine: “*The physician did not tell you how to use; the pharmacist did not know how to use. Buy and swallow*” (FG2).

##### Subtheme 2: Leaflets as an Information Source

Most participants responded that they tried to read accompanying consumer medication leaflets. However, they also said the information provided was hard to understand and frightening: “*So much that is written is terrible, it looks like you will get cancer and so on*” (FG4).

According to the participants, large amounts of information in the leaflets indicated that the medicines were well-researched, and that research was performed to increase the sales of the medicine: “*The more that is written, the better the medicine is studied*” (FG2).

Some participants kept the consumer leaflet as instructed and read it when they thought they were experiencing an ADR. After familiarization with possible ADRs described in the leaflet, participants admitted that they adapted the information to themselves and decided how to respond based on the information (e.g., whether to discontinue treatment): “*You pick up that leaflet and you look for information about what is happening to you and what could happen, and what the consequences are. You read everything, then you know*” (FG2).

#### 3.2.3. Theme 3: Knowledge about ADR Reporting 

A few respondents reported that even if they wanted to report an ADR, they were not familiar with the reporting procedure: “*We don’t know who to tell*” (FG3). Those that read the leaflets were aware that contact details were provided for ADR reporting. However, they did not believe that if they reported the ADR it would affect the frequency of adverse events and thus did not make a report: “*We threw up our hands, and that’s all*” (FG2); “*I don’t believe someone could take care* (of the ADRs)*; as we see now, there are bigger problems in public, and no one is paying attention. And if you are feeling dizzy or vomiting*…” (FG 2).

#### 3.2.4. Theme 4: Attitudes towards ADR Reporting

Even though most participants had experienced at least one ADR, they admitted that there was no one to whom they could report medication-related issues. Most participants thought that their ADRs were not important to anyone and no one in the healthcare system cared about them: “*And who to tell? And who cares here?*” (FG3). During the discussion, it was also mentioned that there was no point in reporting ADRs that had already been resolved: “*Who would you talk to if it’s (the ADR) already over?*” (FG1).

#### 3.2.5. Theme 5: Benefits of ADR Reporting

Participants reported that they experienced personal and altruism-associated benefits after ADR reporting: “The physician will not prescribe that medication anymore for me” (FG1); “Maybe if my reaction was very strong, the doctor will also consider other patients” (FG1).

#### 3.2.6. Theme 6: Barriers to ADR Reporting

##### Subtheme 1: Lack of Time to Report ADRs

A major concern outlined by participants was the lack of time during a consultation to discuss ADRs and receive more information from physicians. The physicians were occupied with incoming patients. Time for consultation was very limited and used for other necessary administrative tasks: “*Too little time for them (physicians) to say more. We rush and rush*” (FG2); “*When he (physician) starts asking questions, he will finish the job in two days...*” (FG1); “*Physician has twenty minutes. What can he do?*” (FG3). The same lack of time was mentioned when discussing pharmacists: “*The pharmacist also does not have time to explain*” (FG2).

##### Subtheme 2: Lack of Interest from Physicians and Pharmacists

Physicians and pharmacists were considered authorities, and participants believed that their lack of interest in patients’ health during consultations discouraged patients from talking about additional topics, such as ADRs: “*If you say to the physician that this hurts me, and he does not examine you, nor send you anywhere*” (FG1); “*Sometimes, a physician doesn’t say a single sentence or phrase about health*” (FG2).

##### Subtheme 3: Low Physician and Pharmacist Motivation 

Some respondents highlighted that physicians very often lacked motivation and ambition in their work, especially those who worked in smaller cities. Collection and reporting of ADRs increases workload. They indicated that physicians and pharmacist were interested in performing their work as quickly as possible, without extra inputs from additional tasks: “*Those working in Vilnius and Kaunas* (the largest cities in Lithuania) *have ambition, but what is the ambition here* (i.e., in a smaller city)? *To work from eight to five. Will he ask you for side effects? If he starts asking everyone here, it will be two days before he can leave work*” (FG1).

##### Subtheme 4: Lack of Education

Lack of knowledge among physicians and pharmacists was given as another possible barrier to effective ADR monitoring and reporting. Some participants indicated that continuous learning was part of pharmacists’ and physicians’ work. However, a lack of time was mentioned as the main reason for the lack of continuous learning: “*First, there should be seminars for pharmacists and physicians, but they have no time for them*” (FG2); “*They (physicians and pharmacists) must be interested. They are there to do their work*” (FG2).

##### Subtheme 5: Physicians’ Attitudes

The physician’s attitude was also indicated as a barrier to ADR reporting. One participant reported that their physician discouraged their search for information, and there was a sense of competition regarding the amount of information the participant presented: “*I informed the family doctor that I was facing this or that problem. She said, treat yourself then. She was resentful*” (FG3).

##### Subtheme 6: Nocebo Effect

One participant reported that a physician discouraged them from reading about ADRs in the leaflets that accompanied their medicine. The participant sensed that the physician wanted to increase their confidence about taking the medicine: “*Well, regarding the information about side effects, the physician said not to pay attention. We’ve told you the benefits and that taking it will be beneficial. They have to write what is written there, and that’s it*” (FG2).

##### Subtheme 7: Lack of a Proper Reporting System

Participants identified the lack of a proper reporting system as a barrier to ADR reporting. However, a major barrier was the lack of a common system between physicians and pharmacists: “*Now every specialist has their own file. Let’s say I go to a neuropathologist, he pulls out his own file, ear doctor takes own file, another file—tooth doctor. No one understands*” (FG2). 

## 4. Discussion

All participants in this study experienced what they perceived to be suspected ADRs associated with medicines they used. The medicines were provided over-the-counter or were prescribed. Irrespective of the ADR severity, participants did not report any of these experiences. Consistent with the findings of other studies, the ADR experiences described by the participants were identified in relation to the timing of the reactions [18,19,20]. Participants also tried to de-challenge or re-challenge the medicine in use, to investigate possible ADRs using an Internet search, to read leaflets or discuss with friends or relatives, some of whom were healthcare professionals. Similarly, as studies performed in Malaysia and the United Kingdom found, our findings suggested that participants did not have proper knowledge about the possible ADRs associated with their medicines [18,19,20]. However, when an ADR occurred, the patients wanted more information and searched for it using a variety of methods. 

In this study, most participants reported that they obtained information about their medicines from a variety of sources (e.g., the Internet and mass media). However, unlike the findings of other studies, leaflets and pharmacists were the primary sources used [20]. Most respondents indicated that leaflets were their first choice as an information source about ADRs. Participants read the leaflets after medicines were purchased; the leaflets were also kept for future reference. In contrast, other studies found that multi-folded small shrift printed leaflets were too complex and hard to understand [21]. However, in the present study, the choice of leaflets as the first source of ADR information was not entirely voluntary. If information about medicines was received from pharmacists, the possible ADRs associated with these medicines were never mentioned by pharmacists nor by physicians.

No participants reported ADRs when they occurred. The main barriers to reporting were a lack of awareness about opportunities for direct consumer reporting, the relationship between the patient and physician, and attitudes towards ADR reporting. 

The findings regarding poor awareness about available reporting systems, and uncertainty about what should be reported and to whom, were consistent with those of previous studies [18,22,23,24,25]. Public awareness of patient reporting schemes has always been low in many countries [18,22,23,24,25]. In many cases, low awareness is attributed to a lack of publicity campaigns. Information indicating that ADRs can be reported in Lithuania is present in leaflets, on the SMCA webpage, and on social media pages (Facebook, Twitter, etc.). Some mass media campaigns about ADR reporting have also been implemented, but only in recent years. However, in general, there is limited information available to the general patient population about the availability of direct patient reporting.

A key finding of the current study was that the relationship between patient and physician was a barrier to the reporting of suspected ADRs. During the FG discussions, pharmacists were indicated as a primary source for information about medicines, while physicians mostly provided information about illnesses. However, patients expected that the physicians would inform them about possible ADRs. One study found that patients tend to report ADRs to physicians because they require confirmation or recognition of the adverse event. Insufficient communication between patients and physicians acts as a barrier to the reporting of suspected ADRs [26]. Our results suggested that physician ignorance and apathy about previously reported ADRs generally stops patients from taking any other actions concerning ADRs. Instead of turning to other professionals or institutions, patients rely on information found on the Internet, in leaflets, and their own knowledge.

Partly because of the poor communication with physicians and their failure to take patient reports seriously, patients had a pessimistic attitude towards the need for and benefits of reporting in general. Only a few patients mentioned motivating factors (e.g., personal or altruistic). Van Hunsel et al. found that over 90% of patients who reported ADRs were motivated by preventing harm to others [27]. However, altruism and personal motivation were not strongly expressed by patients in this study. Our findings suggested that neither altruism nor personal reasons were sufficiently important factors in the non-reporting population.

Several studies provided essential data indicating that the active pharmacovigilance approach implemented in healthcare can significantly increase the number of ADR reports [28,29]. Active calls to patients or reminder messages successfully helped to draw consumers’ attention to possible ADRs that might be mistakenly assigned to illnesses instead of medicines in use. Considering that the number of reported ADRs increased at certain times, an active approach needs to be considered, at least for some medicine groups [29,30]. Active pharmacovigilance might help develop consumer habits of reporting ADRs in the presence of spontaneous pharmacovigilance. The authors should discuss these results and how they can be interpreted from the perspective of previous studies and the working hypotheses. The findings and their implications should be discussed in the broadest context possible. Future research directions may also be highlighted.

## 5. Conclusions

We found that respondents lacked knowledge of possible ADRs associated with their medications. However, they were able to describe the ADRs, provide examples, and identify ADRs. Despite possessing the ability to identify them, no respondents reported ADRs. These findings suggest that poor communication between patients and physicians acted as the main barrier in the under-reporting population. Altruism and personal benefits did not motivate participants to report suspected ADRs. The key issues related to the ineffective dissemination of information about ADRs were similar to those found in Lithuania prior to 2010 [31]. Physicians’ increased workloads, time-consuming documentation, and short consultation times were the main factors indicated by patients as affecting open and honest communication. Physicians are in a position to educate patients about their medicines. However, this study found that there is a lack of clearly set standards and guidelines regarding the flow of communication between consumers, physicians, and pharmacists. Therefore, the inclusion of active pharmacovigilance should be considered.

## Figures and Tables

**Table 1:** Characteristics of participants in focus groups (FGs).

FG	Age in Years (Min–Max)	Characteristic	Frequency	Total
FG 1	32–82	Male	3	8
		Female	5	
FG 2	70–90	Male	4	14
		Female	10	
FG 3	58–88	Male	2	12
		Female	10	
FG 4	29–64	Male	4	8
		Female	4	
			Total	42

## Data Availability

The data presented in this study are available upon request from the corresponding author. The data are not publicly available due to our confidentiality policy.

## References

[1] Inch J., Watson M.C., Anakwe-Umeh S. (2012). Patient versus healthcare professional spontaneous adverse drug reaction reporting: A systematic review. Drug Saf..

[2] European Parliament (2010). Directive 2010/84/EU of the European Parliament and of the Council of 15 December 2010 Amending, as Regards Pharmacovigilance, Directive 2001/83/EC on the Community Code Relating to Medicinal Products for Human Use Text with EEA Relevance.

[3] Blenkinsopp A., Wilkie P., Wang M., Routledge P.A. (2007). Patient reporting of suspected adverse drug reactions: A review of published literature and international experience. Br. J. Clin. Pharmacol..

[4] Van Hunsel F., Härmark L., Pal S., Olsson S., van Grootheest K. (2012). Experiences with adverse drug reaction reporting by patients: An 11-country survey. Drug Saf..

[5] Anderson C., Krska J., Murphy E., Avery A., Yellow Card Study C. (2011). The importance of direct patient reporting of suspected adverse drug reactions: A patient perspective. Br. J. Clin. Pharmacol..

[6] Hazell L., Cornelius V., Hannaford P., Shakir S., Avery A.J. (2013). How do patients contribute to signal detection? A retrospective analysis of spontaneous reporting of adverse drug reactions in the UK’s Yellow Card Scheme. Drug Saf..

[7] Shamseer L., Moher D., Clarke M., Ghersi D., Liberati A., Petticrew M., Shekelle P., Stewart L.A., PRISMA-P Group (2015). Preferred reporting items for systematic review and meta-analysis protocols (PRISMA-P) 2015 statement. Syst. Rev..

[8] Härmark L., van Hunsel F., Grundmark B. (2015). ADR Reporting by the general public: Lessons learnt from the Dutch and Swedish systems. Drug Saf..

[9] 2013 Annual Report (2013). The Drug Control Department under the Government of the Republic of Lithuania. https://www.vvkt.lt/index.php?2417723960.

[10] (2020). The State Medicines Control Agency under the Ministry of Health of the Republic of Lithuania (SMCA). https://www.vvkt.lt/index.php?3341007673.

[11] 2019 Annual Report (2019). The Drug Control Department under the Government of the Republic of Lithuania. https://www.vvkt.lt/index.php?2417723960.

[12] Al Dweik R., Stacey D., Kohen D., Yaya S. (2017). Factors affecting patient reporting of adverse drug reactions: A systematic review. Br. J. Clin. Pharmacol..

[13] Wong L.P. (2008). Focus group discussion: A tool for health and medical research. Singapore Med. J..

[14] Cameron J., Hay I. (2005). Focusing on the focus group. Qualitative Research Methods in Human Geography.

[15] Dawood O.T., Hassali M.A., Saleem F. (2016). A qualitative study exploring medicines use pattern and practice among general public in Malaysia. Pharm. Pract..

[16] Krska J., Jones L., McKinney J., Wilson C. (2011). Medicine safety: Experiences and perceptions of the general public in Liverpool. Pharmacoepidemiol. Drug Saf..

[17] Braun V., Clarke V. (2006). Using thematic analysis in psychology. Qual. Res. Psychol..

[18] Elkalmi R., Hassali M.A., Al-Lela O.Q., Jawad Awadh A.I., Al-Shami A.K., Jamshed S.Q. (2013). Adverse drug reactions reporting: Knowledge and opinion of general public in Penang, Malaysia. J. Pharm. Bioallied Sci..

[19] Jarernsiripornkul N., Patsuree A., Krska J. (2017). Public confidence in ADR identification and their views on ADRreporting: Mixed methods study. Eur. J. Clin. Pharmacol..

[20] Hughes L., Whittlesea C., Luscombe D. (2002). Patients’ knowledge and perceptions of the side-effects of OTC medication. J. Clin. Pharm. Ther..

[21] Raynor D.K., Knapp P. Do Patients See, Read and Retain the New Mandatory Medicines Information Leaflets? Pharmaceutical Journal. February 2000, Online|URI: 20000495. https://www.pharmaceutical-journal.com/do-patients-see-read-and-retain-the-new-mandatory-medicines-information-leaflets/20000495.article?firstPass=false.

[22] Sales I., Aljadhey H., Albogami Y., Mahmoud M.A. (2017). Public awareness and perception toward Adverse Drug Reactions reporting in Riyadh, Saudi Arabia. Saudi Pharm. J..

[23] Avery A.J., Anderson C., Bond C.M., Fortnum H., Gifford A., Hannaford P.C., Hazell L., Krska J., Lee A.J., Mclernon D.J. (2011). Evaluation of patient reporting of adverse drug reactions to the UK ‘Yellow Card Scheme’: Literature review, descriptive and qualitative analyses, and questionnaire surveys. Health Technol. Assess..

[24] Matos C., van Hunsel F., Joaquim J. (2015). Are consumers ready to take part in the Pharmacovigilance System?—A Portuguese preliminary study concerning ADR reporting. Eur. J. Clin. Pharmacol..

[25] Lebanova H., Getov I. (2014). Study of Patients’ Potential as a Source for Spontaneous Reporting Systems in Bulgaria. Pharmacoepidemiol. Drug Saf..

[26] Walji R., Boon H., Barnes J., Austin Z., Welsh S., Baker G.R. (2010). Consumers of natural health products: Natural-born pharmacovigilantes?. BMC Complementary Altern. Med..

[27] Van Hunsel F.P., ten Berge E.A., Borgsteede S.D., van Grootheest K. (2010). What motivates patients to report an adverse drug reaction?. Ann. Pharmacother..

[28] Iannone L.F., Bennardo L., Palleria C., Roberti R., De Sarro C., Naturale M.D., Dastoli S., Donato L., Manti A., Valenti G. (2020). Safety profile of biologic drugs for psoriasis in clinical practice: An Italian prospective pharmacovigilance study. PLoS ONE.

[29] Haas J.S., Klinger E., Marinacci L.X., Brawarsky P., Orav E.J., Schiff G.D., Bates D.W. (2012). Active pharmacovigilance and healthcare utilization. Am. J. Manag. Care.

[30] Huang H.L., Li Y.C., Chou Y.C., Hsieh Y.W., Kuo F., Tsai W.C., Chai S.D., Lin B.Y., Kung P.T., Chuang C.J. (2013). Effects of and satisfaction with short message service reminders for patient medication adherence: A randomized controlled study. BMC Med. Inform. Decis. Mak..

[31] Liseckiene I., Liubarskiene Z., Jacobsen R., Valius L., Norup M. (2008). Do family practitioners in Lithuania inform their patients about adverse effects of common medications?. J. Med. Ethics.

